# Determinants of neuroglobin plasticity highlighted by joint coarse-grained simulations and high pressure crystallography

**DOI:** 10.1038/s41598-017-02097-1

**Published:** 2017-05-12

**Authors:** Nathalie Colloc’h, Sophie Sacquin-Mora, Giovanna Avella, Anne-Claire Dhaussy, Thierry Prangé, Beatrice Vallone, Eric Girard

**Affiliations:** 1 ISTCT CNRS UNICAEN CEA Normandie Univ., CERVOxy team, centre Cyceron, 14000 Caen, France; 2 Laboratoire de Biochimie Théorique, CNRS UPR9080, Institut de Biologie Physico-Chimique, 13 rue Pierre et Marie Curie, 75005 Paris, France; 3 Instituto Pasteur–Fondazione Cenci Bolognetti and Dipartimento di Scienze Biochimiche ‘A. Rossi Fanelli’, Sapienza Università di Roma, 5 piazzale Aldo Moro, 00185 Roma, Italy; 4 CRISTMAT UMR 6508 CNRS ENSICAEN UNICAEN Normandie Univ., 6 bd du Maréchal Juin, 14050 Caen, France; 5 LCRB, UMR 8015 CNRS Université Paris Descartes, 4 avenue de l’Observatoire, 75270 Paris, France; 6 Institut de Biologie Structurale (IBS), Université Grenoble Alpes, CEA, CNRS, 38044 Grenoble, France; 7 BIOGEM Research Institute, Ariano Irpino, Italy

## Abstract

Investigating the effect of pressure sheds light on the dynamics and plasticity of proteins, intrinsically correlated to functional efficiency. Here we detail the structural response to pressure of neuroglobin (Ngb), a hexacoordinate globin likely to be involved in neuroprotection. In murine Ngb, reversible coordination is achieved by repositioning the heme more deeply into a large internal cavity, the “heme sliding mechanism”. Combining high pressure crystallography and coarse-grain simulations on wild type Ngb as well as two mutants, one (V101F) with unaffected and another (F106W) with decreased affinity for CO, we show that Ngb hinges around a rigid mechanical nucleus of five hydrophobic residues (V68, I72, V109, L113, Y137) during its conformational transition induced by gaseous ligand, that the intrinsic flexibility of the F-G loop appears essential to drive the heme sliding mechanism, and that residue Val 101 may act as a sensor of the interaction disruption between the heme and the distal histidine.

## Introduction

Protein motions can be described as discrete transitions between conformational sub-states of different Gibbs free energies^1–3^. Cavities facilitate conformational transitions between sub-states and play a major role in conformational flexibility and domain motions^4–6^. In globins for example, cavities are involved in migration pathways for gaseous ligands (O_2_ and CO), and the plasticity of this inner network is related to the breathing motions of the whole protein^7–9^. As in numerous protein systems^10, 11^, subtle structural modifications in globin structures can lead to large functional perturbations.

Pressure promotes high energy conformers of lower volumes, altering the ensemble of allowed protein conformations, by modifying the Gibbs free energy. As a consequence, it is an ideal tool to study energy landscapes of proteins, conformational fluctuations and breathing motions between sub-states, allowing to explore functional pathways and the structural analysis of conformers that are scarcely populated at ambient pressure^12–17^. As a notable case, ligand binding to a protein induces conformational changes toward a conformer of higher energy, which can be trapped by pressure. In addition, pressure allows to analyze relationships between local rigidity and overall flexibility and to characterize areas adjusted to optimize functional efficiency and that are therefore particularly sensitive to very small structural changes^11, 16, 18–20^.

To investigate the correlation between mechanical properties and functional efficiency in proteins, we selected neuroglobin (Ngb), a hexacoordinated heme protein, which is expressed in the neurons of vertebrates and is involved in neuroprotection in hypoxic conditions and protects brain from stroke^21–26^. The structures of human and murine Ngb present a large internal cavity behind the heme, that is connected to the surface through a tunnel, forming a pathway toward the heme for gaseous ligands (O_2_, CO, NO)^27–29^. Like many recently discovered globins, Ngb is characterized by endogenous heme iron hexacoordination in the absence of external ligands. The structure of murine carbonmonoxy Ngb revealed that to achieve coordination, the heme shifts inside the cavity, inducing a pentacoordinated iron, allowing the ligand to bind^30^. This structural transition, called the “heme-sliding mechanism”, has been confirmed by mutagenesis studies, showing that the two mutants F106W and M144W that hamper the heme sliding inside the cavity, still bind CO but with lower rates and significantly decreased affinities, while the two other mutants V101F and V140W, which allow the heme to slide, bind CO with rates and affinities similar to the wild-type (WT) Ngb^31^. These results point out that the internal cavity, through its plasticity and reshaping, plays a key role in Ngb function. Given the fact that structural data on CO-bound Ngb are available only for the murine protein, this is the most suitable system for investigating the structural plasticity involved in ligand binding and heme displacement.

To provide further insight into Ngb plasticity and the features that drive the heme sliding in this globin, we used a structural approach combining high pressure macromolecular crystallography (HPMX) and coarse-grained Brownian Dynamics simulations on the wild-type Ngb and two mutants in which additional bulk was introduced in the large cavity, showing either unaffected or decreased CO affinity (V101F and F106W respectively)^31^. HPMX allows to determine the most flexible parts of a protein in relation with its internal cavity modifications. The analyses of protein structures determined under high hydrostatic pressure have revealed that the main pressure-induced structural effects occur on the volumes of cavities which are reduced^32–35^. Coarse-grained simulations allow to investigate the mechanical properties of the proteins, in particular those of the frontier residues lining internal cavities, and highlight their mechanical nucleus^36–40^.

## Results

### Crystal structures under ambient pressure (AP) of WT, V101F and F106W Ngb

**Figure 1:**
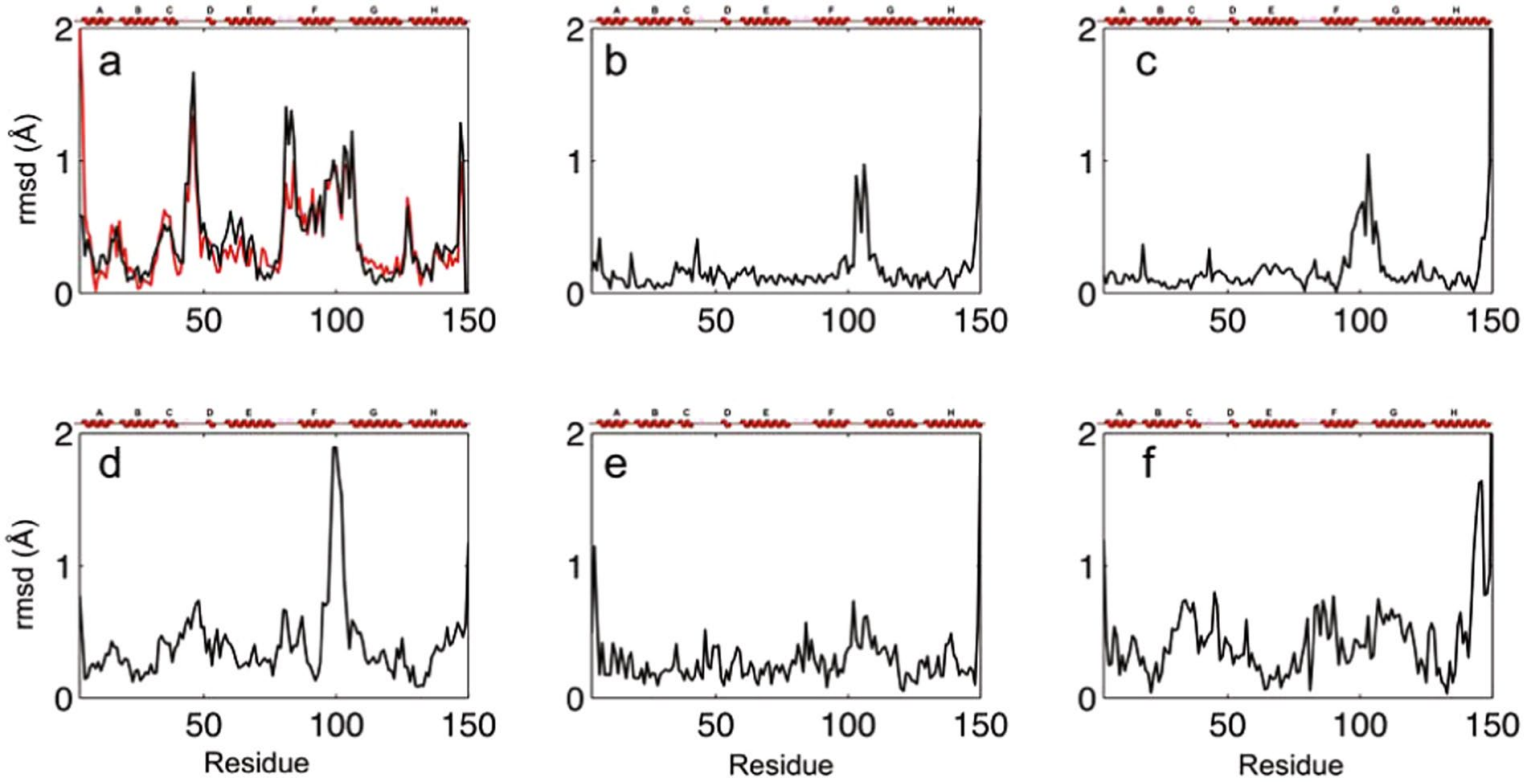
Root mean squares deviations calculated on the Cα chain between different Ngb structures, with secondary structures shown above. (**a)** Carbonmonoxy structure at 100 K (1w92) compared to ferric structure at 100 K (1q1f, in black) or to WT AP (in red). **(b)** V101F AP compared to WT AP. **(c)** F106W AP compared to WT AP. **(d)** WT HP-310 compared to WT AP **(e)** V101F-240 compared to V101F AP. **(f)** F106W HP-310 compared to F106W AP.

**Figure 2:**
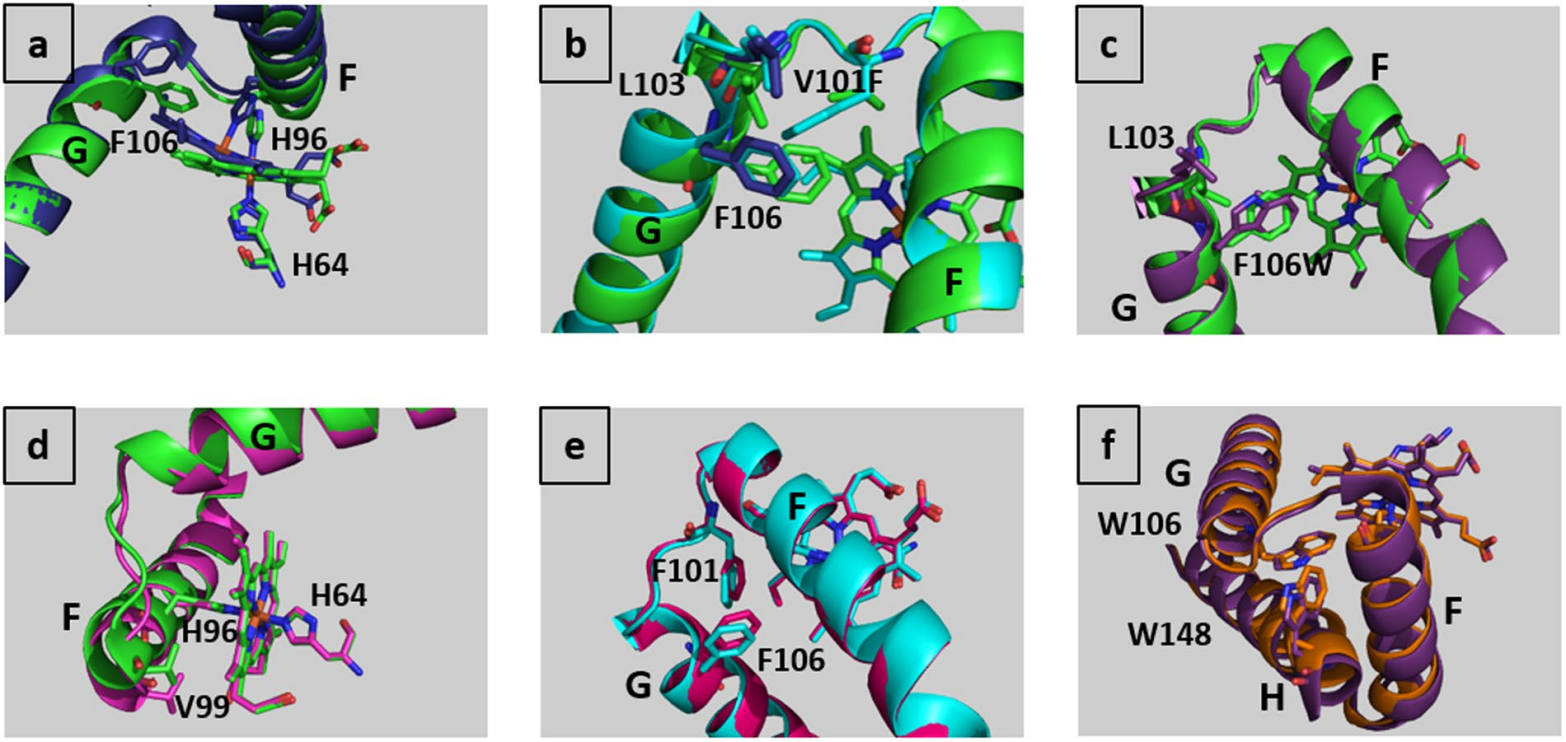
Structures of neuroglobin mutants compared to the wild-type one and high-pressure (HP) structures compared to ambient pressure (AP) structures. For clarity, only helix F, FG loop and helix G are shown in cartoon representation, and the heme, the distal and proximal histidines and notable residues in stick representation. **(a)** Carbonmonoxy Ngb structure (1w92, in dark blue) compared to WT AP (in green). **(b)** V101F AP (in cyan) and WT AP (in green). Leu 103 and Phe 106 from the carbonmonoxy structure (1w92, 100 K) are shown in dark blue for comparison. **(c)** F106W AP (in purple) and WT AP (in green). **(d)** WT HP-310 (in magenta) and WT AP (in green). **(e)** V101F HP-240 (in red) and V101F AP (in cyan). **(f)** F106W HP-310 (in orange) and F106W AP (in purple), helix H is shown in cartoon representation.

Ngb folds like all globins with a bundle of eight helices termed A to H, and a heme hexacoordinated in both ferric and ferrous forms by the imidazole side chain of the distal and proximal histidines (His 64 and His 96 respectively). The loops are named according to their flanking helices, such as “FG loop”. The Ngb WT AP structure at room temperature (Supplementary Table 1) and the 100 K structure of Ngb WT (PDB entry 1q1f) show negligible differences. In these structures, the heme iron is hexa-coordinated by two histidines while in the carbonmonoxy one (CO-Ngb, PDB entry 1w92), the distal histidine is replaced by a gaseous ligand^28, 30, 31^. The CO binding induces, in addition to the heme sliding, a large displacement of Phe 106, a shift of the proximal His 96, the modification of helix F, the FG loop and helix G (Figs 1a, 2a), together with a repositioning of the CD loop^30^.

Residue Val 101, located in the FG loop, points toward the heme on the proximal heme side. Its substitution by a bulkier Phe residue was designed to decrease the volume of the internal cavity. The crystal structure of this mutant was not previously known, consequently the reference structure V101F AP, though to describe the effects of the mutation only, was determined (Supplementary Table 1). The mutation of Val 101 to a Phe residue leads to a shift of the beginning of helix G (Leu 103 – Ser 107) toward the bulk by almost 1 Å (on Cα chain), while helix F is mostly unaffected. In more detail, the side chain of Phe 101 pushes by 1 Å the side chain of Phe 106 toward the position it occupies when the heme has slid into the cavity in the CO-Ngb structure (Figs 1b, 2b). In turn, the side chain of Phe 106 displaces the side chain of Leu 103 to the position it occupied in the CO-Ngb structure. Finally, Leu 103 shifts the Trp 148 and Asp 149 side chains at the C-terminal end of Ngb. The positions of the heme, the proximal and the distal histidines are unaffected by the mutation. Mean B-factors in the mutant are similar to the WT (Supplementary Table 1). Contrary to expectations, the volume of the internal cavity increases by almost 5% upon the Val 101 to Phe mutation (Supplementary Table 2), due to the shift of helix G away from the heme.

The 100 K structure of F106W (PDB entry 4o1t) and its modification compared to 100 K WT Ngb (PDB entry 1q1f) have already been described^31^. The F106W AP structure at room temperature described here (Supplementary Table 1) and the 100 K structure 4o1t show only minor differences in the very flexible CD loop. In the CO-Ngb structure, Phe 106 is the residue mostly affected by the shift of the heme inside the cavity^30^. Its mutation to a Trp hampers heme displacement, leading to low CO affinity^31^. The mutation of the Phe 106 to a Trp leads to a shift of the segment Lys 95 – Ser 107 (termed extended FG loop) toward the bulk (Figs 1c, 2c). In particular, the side chain of Leu 103 is pushed towards the bulk by the side chain of Trp 106. The shift of Leu 103 leads to a shift of the side chain of Trp 148, located at the end of helix G, and to repositioning of the C-terminal Gly 150. Mean B-factors in the mutant are similar to the WT (Supplementary Table 1). The volume of the internal cavity is reduced by 4.6% upon the F to W mutation as expected (Supplementary Table 2).

### Crystal structures under high hydrostatic pressure (HP) of WT, V101F and F106W Ngb

Ngb crystals of WT, V101F and F106W diffract up to 300–350 MPa, but they slowly degrade, and completely lost their diffraction properties above 400–450 MPa. As previously observed^34, 41, 42^, analyses of the compressibility curves (the metric dependencies of the crystal cell parameters to the applied pressure) indicate that the unit cell volume shrinks by 3.1% (WT at 310 MPa), 4% (V101F at 240 MPa), and 5.9% (F106W at 310 MPa), with respect to the ambient pressure unit-cell (Supplementary Fig. 1).

*WT Ngb at 270 and 310* *MPa* - The areas mainly affected by pressure in the WT HP-310 structure are the CD loop (Gln 43 – Ser 50), the end of helix H (Arg 146 - Asp 149), and notably the extended FG loop (segment Lys 95 – Ser 107) which is displaced by almost 2 Å toward the bulk (Figs 1d, 2d), a shift clearly highlighted by the radial deviation analysis (Supplementary Fig. 2). The heme position, and the two distal and proximal histidines are not affected by pressure.

**Figure 3:**
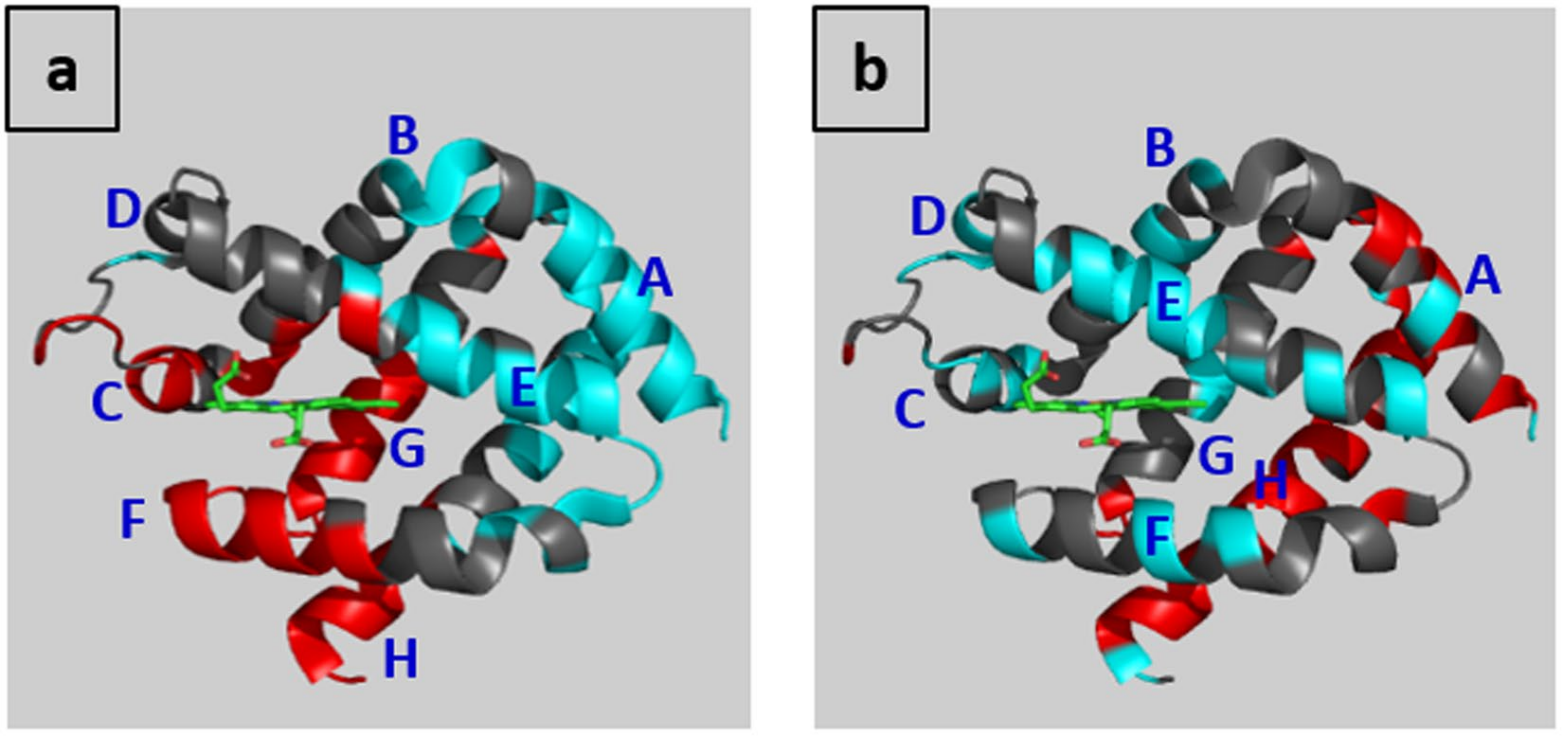
B-factor differences between Ngb HP and AP structures. Ngb shown in cartoon representation with the heme shown in stick representation. **(a)** B-factor differences between WT HP-270 and WT AP (on average 2 Å^2^). Residues are colored according to the B-factor differences, in red if B-factor differences are higher than 4 Å^2^, in cyan if B-factor differences have negative values, in grey otherwise. **(b)** B-factor differences between F106W HP-280 and F106W AP (on average 10 Å^2^). Residues are colored according to the B-factor differences, in red if B-factor differences are higher than 15 Å^2^, in cyan if B-factor differences are lower than 5 Å^2^, in grey otherwise.

The protein mean B-factors increase slightly with pressure (Supplementary Table 1), however masking an uneven distribution of the variations. The residues whose B-factors significantly increase with pressure are mainly located close to the heme while the residues whose B-factors decrease with pressure are mainly located on the back of the internal cavity (Fig. 3a). Between the WT HP-270 and the WT HP-310 structures, there is no clustering of the B-factor differences (Supplementary Fig. 3a), indicating that the uneven distribution of B-factor between the WT AP and the WT HP-270 structures would not be due to a crystal packing effect. Interestingly, in the WT HP-270 structure, the extended FG loop presents increased B-factors (10 Å^2^ or more) and residual densities, indicating that at 270 MPa, the shift has been initiated but could not be modelled.

**Figure 4:**
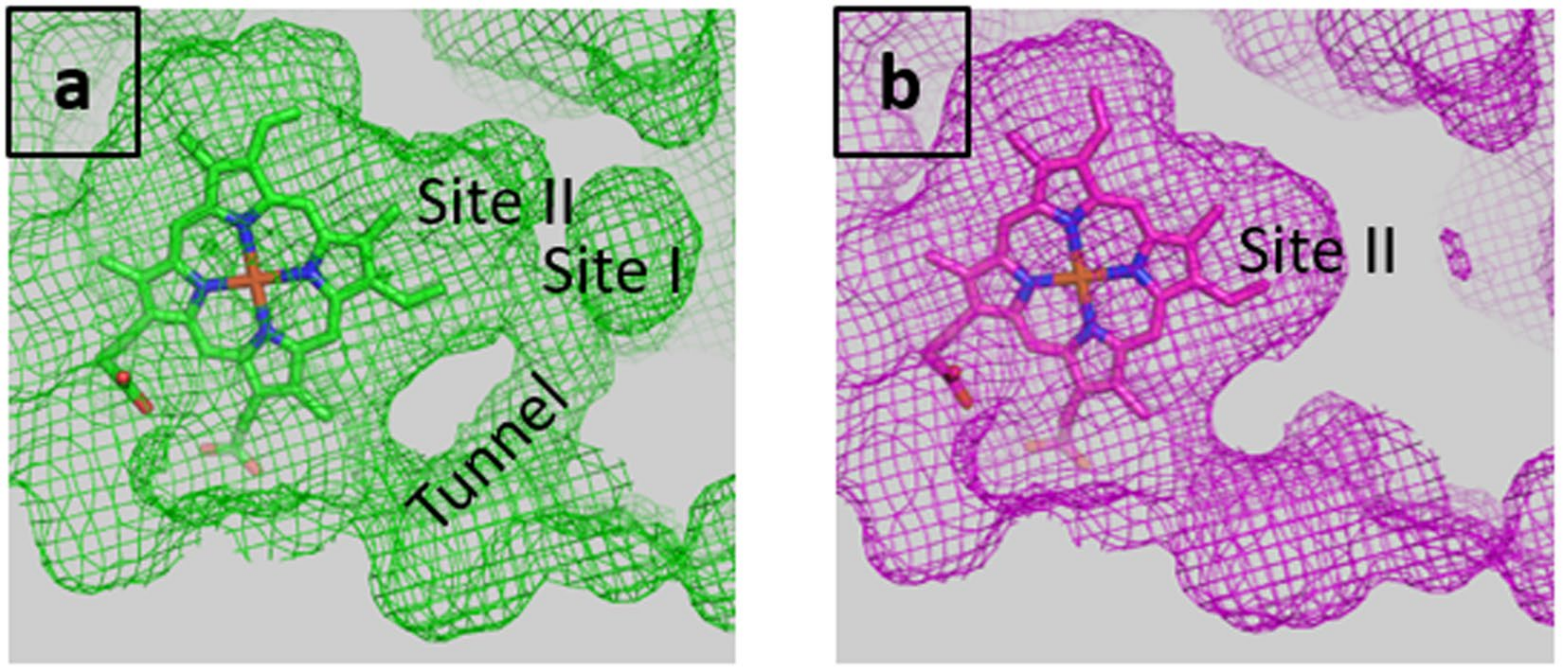
Surface of WT Ngb shown in mesh representation, with the heme shown in stick. **(a)** WT AP. **(b)** WT HP-310.

As expected, the main pressure-induced structural modifications occur in the internal cavity. There is a significant reduction of its volume by 13% at 310 MPa (Supplementary Table 2). Moreover, the tunnel which connects the back of the cavity to the bulk and is likely to be a pathway for gaseous ligands^43^ disappears at 270 MPa (Fig. 4). In the previously determined Ngb structures under pressurized xenon^43, 44^, the large cavity is characterized as two sub-cavities, termed sites I and II, probed by xenon binding. Inside this cavity, sites I and II define a path which connects the protein surface to the heme distal site. Site I is located at the back of the cavity, close to the tunnel, and site II is located between the heme and site I (Fig. 4a). In the WT HP-270 structures, both sites are still present, while, in the WT HP-310 structure, site I disappears while site II remains (Fig. 4b).

*V101F mutant at 150 and 240* *MPa* - The V101F mutant crystal seems to be more sensitive to pressure than the WT, possibly due to the increased cavity volume and to destabilization of internal packing (Supplementary Fig. 1). There are no significant differences between V101F HP-150 and V101F AP. Comparing V101F HP-240 and V101F AP (or V101F HP-150), the main zones affected by pressure are the FG loop and the beginning of helix G (F101 – G110), which are also the segments mainly affected upon mutation, while helix F is not modified (Figs 1e, 2e).

The protein mean B-factors slightly increase between V101F AP and V101F HP-150 and markedly in V101F HP-240 (Supplementary Table 1). This elevation is higher than for the WT Ngb, consistent with the V101F higher sensitivity to pressure. The distribution of the B-factor differences is uniform (Supplementary Fig. 3b).

As for the WT, the volume of the cavity decreased markedly by 25% between V101F AP and HP-240 structures (Supplementary Table 2). This shrinkage is higher than observed in Ngb WT, even though the volumes for this cavity in the high pressure structures are similar. In the V101F HP-240 structure, the tunnel connecting the back of the cavity to the bulk disappears, while both sites I and II are still present, similarly to what is observed in the structure of WT HP-270 (data not shown).

*F106W mutant at 280 and 310* *MPa* - The F106W mutant crystal also seems to be more sensitive to pressure than the WT, but more resilient than the V101F mutant (Supplementary Fig. 1). There are no major differences between F106W HP-280 and F106W HP-310. Between F106W HP-310 and F106W AP, the main segments affected by pressure are the BC and CD loops, the N-terminal segment of helices F and G, and especially the second half of helix H which is shifted by more than 1 Å toward helix G which carries the F106W mutation (Figs 1f, 2f).

The protein mean B-factors are considerably raised by pressure (Supplementary Table 1), which could explain why this mutant is more sensitive to pressure than WT Ngb. The large increase in average in B-factors hides some heterogeneities in the distribution of the B-factors variations. The residues whose B-factors present a relevant increase with pressure (more than 15 Å^2^) are mostly located at the back of the protein, while the residues whose B-factors are moderately increased by pressure (less than 5 Å^2^) are mainly located close to the heme (Fig. 3b). The effect of pressure seems to be the opposite of what was previously observed in WT Ngb under pressure.

As for the WT Ngb, the main pressure-induced structural modifications occurred on the internal cavity. There is a shrinkage of its volume between the HP-310 and AP structures by 8% (Supplementary Table 2). In particular, the tunnel disappears already at 280 MPa and site I disappears at 310 MPa, while site II remains insensitive to pressure (data not shown).

### Mechanical properties of frontier residues

Similar to what has been previously observed for hemoproteins^36^ and several members of the globin family^45–47^, the Ngb WT mechanical profile reflects the typical α-helical globin fold, with α-helices appearing as grouped rigidity peaks along the protein sequence (Supplementary Fig. 4).

**Figure 5:**
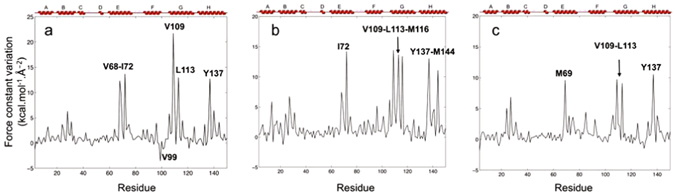
Force constant variation in kcal.mol^−1^.Å^2^ (1 kcal.mol^−1^.Å^−2^ = 0.7 N.m^−1^) along the protein sequence. **(a)** when changing from ambient pressure to 310 MPa in the WT, **(b)** when changing from ambient pressure to 240 MPa in the V101F mutant, **(c)** when changing from ambient pressure to 310 MPa in the F106W mutant.

The force constant variations between Ngb WT HP-310 and WT AP show that HP induces a slight general increase of the protein rigidity with very heterogeneous variations along the protein sequence. Five residues undergo an important increase of their force constant, Val 68, Ile 72, Val 109, Leu 113, and Tyr 137. These five residues are located at the center of the internal globin cavity network, close to the heme and form a mechanical nucleus which is rigidified by pressure. Val 99 on the contrary is the only residue showing a noticeable decrease of its rigidity upon pressure, it belongs to the extended FG loop (Fig. 5a).

Like WT Ngb, both mutants V101F and F106W present a slight general increase of their rigidity when undergoing pressure increase. The force constant variation profiles between V101F HP-240 and V101F AP presents six peaks (Fig. 5b), four of them belong to the mechanical nucleus observed in WT Ngb, and two additional peaks correspond to residues Met 116, which lines an extension of site II only present in this mutant, and Met 144 found in the proximal side of the cavity. The force constant variation profiles between F106W HP-310 and F106W AP presents four peaks (Fig. 5c), three of them correspond to residues belonging to the WT mechanical nucleus, and one corresponds to Met 69, which lies away from the main cavity. None of the mutants show residues with an important decrease in rigidity upon pressure, unlike what was observed for Val 99 in WT Ngb.

## Discussion

Pressure is an efficient tool to explore conformational landscape in proteins, allowing the detection of the most flexible and the most rigid part of proteins and to characterize areas particularly sensitive to very small structural modifications. Combining for the first time high pressure crystallography and coarse-grain simulations applied to Ngb which possesses an internal cavity that undergoes a reshaping upon gas binding, gives access to new and important information about Ngb structural determinants.

HPMX WT Ngb structures reveal the destabilization of the zone close to the heme by pressure with an increase of the B-factors, whereas the zone at the back of the protein is stabilized by pressure with a decrease of the B-factors (Fig. 3a). Notably, the coarse-grain simulations on HP and AP WT structures reveal the existence of a mechanical nucleus composed of five hydrophobic residues (Val 68, Ile 72, Val 109, Leu 113, and Tyr 137) (Fig. 5a), located at the frontier between these two zones. Very interestingly, the previously performed analysis of the globin family using a combination of all-atom molecular dynamics and coarse-grain simulations highlighted the existence of a four residues mechanical nucleus (E11, E15, G8 and G12), whose constituents are essential for controlling ligand migration within the protein^46^. For comparisons between different globins, these residues are indicated according to their position along the α-helices, for example the distal His 64, located at the 7^th^ position of helix E, is named E7. There is thus a very high correlation between the mechanical nucleus revealed by earlier coarse-grained dynamic simulations (E11, E15, G8 and G12) and the residues that present the higher pressure-induced force constant variations with four identical residues Val 68 (E11), Ile 72 (E15), Val 109 (G8) and Leu 113 (G12) and a fifth residue Tyr 137 (H12) which was also shown to occupy a mechanically sensitive position in Ngb even-though this position was not conserved throughout the globin family.

Interestingly, it was shown that Val 68 and Val 109 are two key residues that control heme sliding, affinity for ligand and ligand migration, and their replacement by a bulkier residue slows down the kinetic of ligand binding. Conversely, mutation of Val 68 to a smaller residue (Ala) increases the oxidation rate and slows down nitrite reduction. Moreover, this residue, together with the distal histidine and Phe 28, plays a role in the stabilization of the complex with cyanide and nitrite. It has been hypothesized that the reductase activity of Ngb would be correlated with the heme accessibility of nitrite^48–52^. In Hell’s gate globin I, a bacterial hemoglobin resembling mammalian Ngb^53^, it was shown that ligand migration is controlled by three hydrophobic residues Leu 58, Leu 99 and Tyr 123 which are structurally aligned in Ngb to three residues belonging to the mechanical nucleus, Ile 72, Leu 113 and Tyr 137. Our work highlights these three residues as new key positions to mutate to modulate Ngb dynamics and function.

The use of noble gas pressurized crystallography is a way to characterize hydrophobic cavities^54^ and in recent structures of Ngb under pressurized xenon^43, 44^, it was shown that the xenon atom in site II is lined by the five hydrophobic residues precisely forming the Ngb mechanical nucleus. Moreover, Xe still binds in this site when CO is bound and the heme has slid^43^. This combined HPMX and coarse-grain simulation analysis thus highlights that Ngb hinges around this mechanical nucleus, which is not affected by the heme sliding mechanism, and that this mechanical nucleus, located between the heme and the tunnel, would then control ligand migration to and from the heme.

The tunnel which connects the bulk to site II in the different ferric state structures is drastically reduced in structures of CO-bound WT Ngb and F106W mutant (PDB entries 1w92 and 4o35 respectively)^31, 43^ and disappears in all WT and mutant HP structures (Fig. 4b). Sites I and II are thought to define a path inside the cavity, that was proposed to store oxygen molecules on their way to the heme^7, 43^. Moreover, since the neuroprotective effects of Ngb may be mediated through several mechanisms like oxygen transport, cytochrome C reduction, nitrite reduction, interaction with G proteins, or NO scavenging activity, it has been proposed that a second ligand such as NO could be hosted in the cavity to react with the oxygen bound to the iron^25, 52, 55–59^. Site II could represent a reservoir for NO on the way to the active site. The Ngb HP structures would then correspond to intermediate states where the gaseous ligands migrate toward the heme through the site II while no other gaseous molecules could migrate from the bulk, since both site I and the tunnel have disappeared^60^. Since we have previously shown that nitrous oxide (N_2_O) binds in site I and site II^44^, we can infer that nitrite could fit inside site II, which should not affected by structural modifications occurring during Ngb reductase activity^52^.

CO binding, but not pressure, induces the shift of the heme and the displacement of residues His 96 and Phe 106. However, both CO binding and pressure induce a relevant shift away from the heme of the extended FG loop (Figs 2a, 2d). The Ngb sub-state trapped by pressure, with a shifted F-G loop and a hindered access to the bulk is likely to correspond to a high-energy conformer which would occur during gaseous ligand binding^60^. We can therefore suggest that the shift of helices F and G might drive the heme sliding mechanism since it precedes the heme shift and the His 96 and Phe 106 displacements. This would indicate an intrinsic dynamics of Ngb, at the level of the extended FG loop, where the Val 99 residue which shows and important flexibility upon pressure is located.

The structure of the V101F mutant provides clues about the role of Val 101, the intrinsic flexibility of the extended FG loop, and the very high flexibility of Val 99. Indeed, for this mutant, ligand affinity was slightly increased^31^. Unexpectedly, the introduction of a Phe at position 101 located in FG loop induces the displacement of Phe 106 in the position observed in the CO-Ngb structure^30^, i.e. when gaseous ligand is bound and the heme sliding accomplished. This may explain why V101F mutant showed unaffected CO affinity. Moreover, helix G in the V101F mutant shifts toward the bulk exactly in the same position it occupies in the CO-Ngb structure, while helix F stays as it is in the hexacoordinate structure. The displacement of Phe 106 may then be induced without disruption of the interaction between the distal His 64 and the heme. We would therefore suggest that Val 101, spatially close to the proximal His 96 located at the end of helix F, would act as a sensor of the displacement of His 96 during ligand binding that implies rupture of the distal His 64– heme Fe bond, therefore leading to a transient instability of the heme moiety. Val 101 may then ensure the propagation of the signal toward Phe 106 through helix G. The displacement of the rigid body constituted by the His 96 attached to the heme would be detected by Val 101, inducing the relocation of the highly flexible extended FG loop, so that it accompanies the sliding movement of the heme to stabilize the pentacoordinate intermediate, upon gaseous ligand binding. This would explain why the flexibility of the extended FG loop is necessary to initiate the heme sliding mechanism.

Superposition of the CO-Ngb 1w92 structure to the V101F mutant shows that Phe 101 would clearly collide with the heme in the CO-Ngb structure. Therefore Phe 101 in V101F Ngb has to be displaced upon ligand binding to an unknown position since no CO-Ngb V101F structure has yet been determined. However, unaffected ligand affinity suggests that the energetic cost for heme sliding induced by residue Phe 101 should be comparable to the one induced by Phe 106 in WT Ngb.

The effects of high-pressure on the structure of the F106W mutant shed light on the role of Phe 106 and helix G. This mutant is endowed with an enhanced barrier to heme sliding, as indicated by decreased CO affinity^31^. In this mutant, the mechanical nucleus still coincides with the hinge region, but the whole protein behaves in a reverse way compared to WT-Ngb, with the zone close to the heme stabilized by pressure and the zone at the back of the protein destabilized by pressure (Fig. 4b). The HP structures reveal that the flexibility of the extended FG loop is restrained by the presence of a tryptophan at position 106, with no particular flexibility of Val 99. This is consistent with our hypothesis of a mandatory flexibility of the extended FG loop to initiate heme sliding mechanism and would thus explain the decrease of CO affinity of this mutant.

The previously determined structure of CO-Ngb F106W^31^ (PDB entry 4o35) revealed that the heme can slide inside the cavity and CO can bind as a sixth coordinate, while Trp 106 has mainly flipped toward the solvent. This induces an important repositioning of the end of helix H. The HP structure of the F106W mutant also shows a displacement of the end of helix H, although smaller, suggesting that the F106W HP-310 structure could be an intermediate between the ferric mutant structure and the CO-bound one.

Altogether, our results highlight that Ngb would hinge around a mechanical nucleus (Val 68, Ile 72, Val 109, Leu 113, Tyr 137) during its conformational transition induced by gaseous ligand, that the intrinsic flexibility of the extended FG loop (segment Lys 95 – Ser 107) would be necessary to initiate the heme sliding mechanism, and that the residue Val 101 might act as a sensor of the initial displacement of the heme and proximal histidine, brought about by rupture of the heme bond with His 64. The combined analysis of experimental and theoretical works, HPMX and coarse-grain simulations, sheds light on the balance between a rigid mechanical nucleus and a flexible extended FG loop for an optimal functional efficiency of Ngb. The analysis of the structural plasticity, perturbations and states induced by high pressure on WT Ngb and its mutants unveiled how the highly conserved globin mechanical nucleus can support reversible coordination and heme positioning.

## Methods

### HPMX experiments

Crystals of WT, V101F and F106W Ngb were obtained by the hanging drop technique at 293 K, using a 1:1 mixture of protein (10 mg ml^−1^) and reservoir solution [1.6 *M* ammonium sulfate, 0.1 *M* MES pH 6.5, 10%(*v/v*) dioxane]. Rhombohedral Ngb crystals were loaded into a diamond anvil cell (DAC), as previously described in^61, 62^. The solution used as the compression medium consisted of the mother liquor with a 10% higher concentration of ammonium sulfate.

Diffraction data were recorded at room temperature on the CRISTAL beamline at the synchrotron SOLEIL (Saint-Aubin, France) for wild-type Ngb and on the ID09 beamline at the ESRF synchrotron (Grenoble, France) for the two mutants, at wavelengths λ = 0.4540 Å and 0.4104 Å respectively. Detectors were a Rayonix SX165 on CRISTAL and a Marresearch Mar555 flatpanel on ID09. The pressure within the DAC compression chamber was monitored through the pressure-dependent fluorescence of a ruby chip used as an internal probe^61^. Exposure times were 30 s. and 1 s. per frame respectively on CRISTAL and ID09 for an oscillation angle of 1°. Only one crystal was used for each data set, thanks to the large aperture DAC, specifically designed for HPMX^62, 63^. During the data collections, the crystal was translated in the beam every 10° of rotation to limit the crystal degradation by irradiating fresh portions of the crystal^63^. Crystallographic structures of WT Ngb and F106W mutant were previously determined using standard cryo-crystallography^28, 31^. However, since HPMX data are collected at room temperature, we also collected reference data sets at ambient pressure in the same sample environment, to minimize potential experimental bias and to provide accurate and meaningful comparisons of high pressure effects.

All data sets were indexed and integrated using *XDS* ^64^. The integrated intensities were scaled and merged using *SCALA* and *TRUNCATE* and the structures were solved by molecular replacement (MR). In order to minimize any bias due to the starting model, MR’s were performed by *PHASER* using a poly-Ala chain of Ngb followed by automatic side chain building using *BUCCANEER*. The structure refinements were performed using *REFMAC*. All these programs are part of the CCP4 package^65^. The graphic program *Coot* ^66^ was used to visualize | 2F_obs_ – F_calc_ | and | F_obs_ – F_calc_ | electron density maps and for manual rebuilding during refinement steps.

Crystal-cell compressibility curves (i.e. unit-cell parameter changes *versus* the pressure) were determined for WT Ngb and the two mutants (Supplementary Fig. 1). Three data sets were collected for WT Ngb, at ambient pressure (WT AP, resolution 2 Å), at 270 MPa (WT HP-270, resolution 1.90 Å) and at 310 MPa (WT HP-310, resolution 2.05 Å). Three data sets were collected for V101F Ngb, at ambient pressure (V101F AP, resolution 2 Å), at 150 MPa (V101F HP-150, resolution 1.95 Å) and at 240 MPa (V101F HP-240, resolution 2.40 Å). Three data sets were collected for F106W Ngb, at ambient pressure (F106W AP, resolution 1.75 Å), at 280 MPa (F106W HP-280, resolution 2.15 Å) and at 310 MPa (F106W HP-310, resolution 2.10 Å). All crystals belong to the rhombohedral space-group H32.

The r.m.s.d’s and B-factors differences between structures were analyzed using programs from CCP4 package^65^, and radial expansions/contractions between structures were calculated by the *INFLATE* program (T. Prangé, personal communication). Volumes of the internal cavities were calculated using *CASTp* with a probe radius of 1.4 Å^67^. Figures 2, 3 and 4 and Fig. S3 have been prepared using *PYMOL* (DeLano Scientific, CA, USA).

A summary of data collection and refinements statistics is reported in Supplementary Table 1 and the r.m.s. deviation calculated on Cα chains between different Ngb structures are reported on Supplementary Table 3. All structures were deposited with the Protein Data Bank.

### Computer simulations

Coarse-grained Brownian Dynamics simulations using the ProPHet (Probing Protein Heterogeneity) program^37^ have been carried out using the ambient and high-pressure crystal structures of WT Ngb and the two V101F and F106W mutants as starting conformations. The simulations use a reduced protein model, in which each amino acid is represented by one pseudo-atom located at the C_α_ position, and either one or two (for larger residues) pseudo-atoms replacing the side chain (with the exception of Gly)^68^. Interactions between the pseudo-atoms are treated according to the standard elastic network model^69^. The elastic network model is a simplification of the heterogeneity of internal protein forces, as all pseudo-atoms lying closer than 9 Å are joined with quadratic springs having a force constant of 0.6 kcal mol^−1^ Å^−2^. Springs are assumed to be relaxed in the starting conformation of the protein. Following earlier studies, which showed how ligands as large as a heme group actually had little influence on calculated force constants^36, 70^, we chose not to include the prosthetic heme group in the protein representation. The simulations use an implicit solvent representation via the diffusion and random displacement terms in the equation of motion^71^, and hydrodynamic interactions are included through the diffusion tensor^72^. Further details regarding the simulation procedure can be found in^70, 73^.

The Brownian dynamics simulations have been performed with 200,000 steps at an interval of 10 fs and at a temperature of 300 K. Effective force constants for displacing each particle *i* are then calculated as

$${k}_{i}=\frac{3{k}_{B}T}{\langle {({d}_{i}-\langle {d}_{i}\rangle )}^{2}\rangle }$$

where the brackets indicate the average taken over the whole simulation, *k*_*B*_ is the Boltzmann constant, and *d*_*i*_ is the average distance of particle *i* from the other particles *j* in the protein, excluding the pseudoatoms, which belong to the same residue *m* to which also particle *i* belongs. The distances between the C_α_ pseudoatom of residue *m* and the C_α_ pseudoatoms of the adjacent residues *m + 1* and m − 1 are not included in the average. The force constant associated with each residue *m* is taken to be the average of the force constants calculated according to equation 1 for each of the pseudoatoms *i* forming the residue. Within this framework, the mechanical properties of a protein are described at the residue level by its “rigidity profile”, that is, by the ordered sequence of the force constants (in kcal mol^−1^ Å^−2^) calculated for each residue.

### Data availability

The atomic coordinates and structure factors have been deposited in the Protein Data Bank, Ngb WT AP 5eet, WT HP-270 5eoh, WT HP-310 5eqm, Ngb V101F AP 5eu2, V101F HP-150 5ev5, V101F HP-240 5EY5, Ngb F106W AP 5eys, F106W HP-280 5f0b, F106W HP-310 5f2a.

## Electronic supplementary material

Supplementary information
