# Supplementary material for: Determinants of neuroglobin plasticity highlighted by joint coarse-grained simulations and high pressure crystallography
Source: Sci Rep. 2017 May 12;7:1858. doi: 10.1038/s41598-017-02097-1 (PMC5431840; doi:10.1038/s41598-017-02097-1)
Supplement: Supplementary file 1 — Supplementary information [file 41598_2017_2097_MOESM1_ESM.pdf]

Supplementary information

Determinants of neuroglobin plasticity highlighted by joint coarse-grained simulations and high pressure crystallography

Nathalie Colloc'h[1,*], Sophie Sacquin-Mora[2], Giovanna Avella[3,†], Anne-Claire Dhaussy[4], Thierry Prangé[5], Beatrice Vallone[3], Eric Girard[6,*]

[1]ISTCT CNRS UNICAEN CEA Normandie Univ., CERVOxy team, centre Cyceron, 14000 Caen, France

[2]Laboratoire de Biochimie Théorique, CNRS UPR9080, Institut de Biologie Physico-Chimique, 13 rue Pierre et Marie Curie, 75005 Paris, France

[3]Instituto Pasteur–Fondazione Cenci Bolognetti and Dipartimento di Scienze Biochimiche 'A. Rossi Fanelli', Sapienza Università di Roma, 5 piazzale Aldo Moro, 00185 Roma, Italy.

[4]CRISTMAT UMR 6508 CNRS ENSICAEN UNICAEN Normandie Univ., 6 bd du Maréchal Juin, 14050 Caen, France.

[5]LCRB, UMR 8015 CNRS Université Paris Descartes, 4 avenue de l'Observatoire, 75270 Paris, France.

[6]Institut de Biologie Structurale (IBS), Université Grenoble Alpes, CEA, CNRS, 38044 Grenoble, France.

[†] Present address: BIOGEM Research Institute.

[*]Correspondence should be addressed to N.C. (colloch@cyceron.fr) and E.G. (eric.girard@ibs.fr).

Supplementary Table 1. Data collection and refinement statictics.

Ngb	WT AP	WT HP-270	WT HP-310	V101F AP	V101F HP-150	V101F HP-240	F106W AP	F106W HP-280	F106W HP-310
Pressure (MPa)	0.1	270	310	0.1	150	240	0.1	280	310
Resolution (Å)	2.00	1.90	2.05	2.00	1.95	2.40	1.75	2.15	2.10
Cell									
a (Å)	88.32	87.56	87.37	89.02	87.93	87.2	88.78	86.21	85.99
c (Å)	114.46	113.64	113.28	113.88	113.7	113.9	114.31	114.46	114.58
Cell volume (Å ^{3})	773219	754525	748874	781544	761317	750455	780270	736715	733728
Completeness (%)	98.7 (99.7)	99.8 (99.7)	97.6 (97.8)	97.7(97.9)	99.4 (99.7)	100 (100)	99.8 (99.8)	100 (100)	99.5 (99.9)
Multiplicity	4 (4.1)	4.7 (4.7)	5 (5)	5.3 (5.4)	4.1 (4.2)	5 (5.1)	4.5 (4.6)	8.8 (8.9)	4.2 (4.2)
Unique reflections	11623 (1685)	13380 (1908)	10340 (1509)	11681 (1669)	12433 (1807)	6714 (966)	17681 (2551)	9151 (1318)	9673 (1396)
R merge (%)	10.4 (47.9)	9.3 (47.3)	11.2 (54.1)	12.9 (87.2)	8.9 (72.9)	18.3 (78.1)	7.8 (68.8)	15.1 (86.3)	12.6 (86.6)
Mean I/σ(I)	10.2 (3.3)	10.3 (3.3)	9.8 (3.2)	9.4 (2)	10.2 (1.8)	4.9 (1.8)	10.8 (1.7)	10.2 (3.3)	7.4 (1.6)
Rwork (%)	14.31	16.86	17.15	15.52	15.29	14.37	15.38	17.06	16.45
Rfree (%)	18.05	22.14	23.71	20.9	20.27	26.03	18.65	23.8	26.02
Mean standard deviation from ideality									
Length (Å)	0.019	0.02	0.023	0.021	0.02	0.033	0.015	0.027	0.028
Angle (°)	1.879	2.01	2.219	1.972	2	2.922	1.685	2.451	2.602
Mean B factors (Å ^{2})									
Protein	30.4	32.1	33.7	28.9	31.9	39.3	30.9	42.1	41.1
Heme	19.3	21.9	21.3	21.8	21.7	26	19.3	20.4	21
Water	38.3	37.7	35.6	40.3	41	35.8	42.6	40.2	41.9
Number water	76	81	71	76	76	51	98	73	85
PDB code	5eet	5eoh	5eqm	5eu2	5ev5	5ey5	5eus	5f0b	5f2a

Data between parentheses correspond to the highest resolution shell. *Rsym = ∑h.k.l ∑i |Ii(h.k.l) - <Ii(h.k.l)>|/ ∑h.k.l ∑i Ii(h.k.l), where Ii(h.k.l) is the intensity of observed reflections and <Ii (h.k.l)> the weighted mean of all observations after rejection of outliers. §Rwork = ∑|Fo|-|Fc|/ ∑|Fo|; indicates accuracy of the model. #Rfree is a cross validation residual using 5% of the native data which were randomly chosen and excluded from the refinement.

Supplementary Table 2. Internal cavity volumes (in Å^3)

WT AP	WT HP-270	WT HP-310	V101F AP	V101F HP-150	V101F HP-240	F106W AP	F106W HP-280	F106W HP-310
911	829	790	956	944	713	869	830	800

Supplementary Table 3. Root mean squares deviation (in Å) calculated on the Cα chain between different Ngb structures.

	1W92	V101F AP	F106W AP*	WT HP-270	WT HP-310
WT AP	0.43	0.17	0.17	0.24	0.41

	V101F HP-150	V101F HP-240
V101F AP	0.06	0.27

.	F106W HP-280	F106W-HP-310
F106W AP	0.42	0.45

*Without the C-ter Gly residue

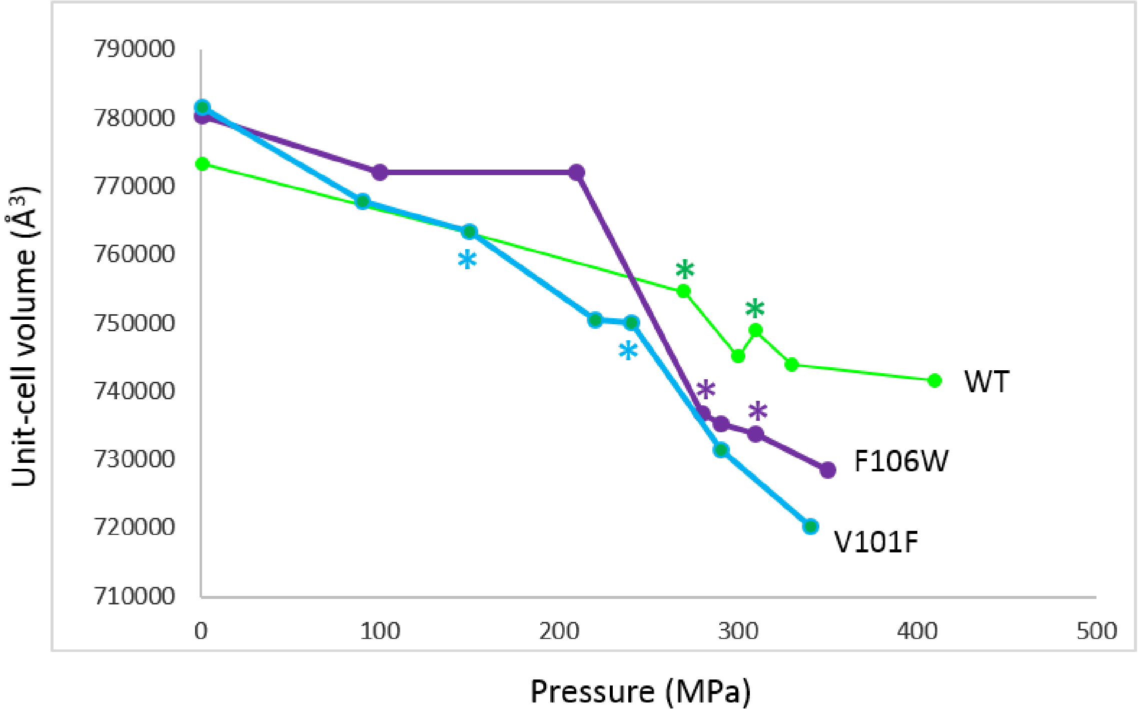

Supplementary Figure 1. Unit-cell compressibility curves for Ngb WT (in green), V101F (in cyan) and F106W (in purple). The pressure corresponding to the different collected data sets are indicated by stars.

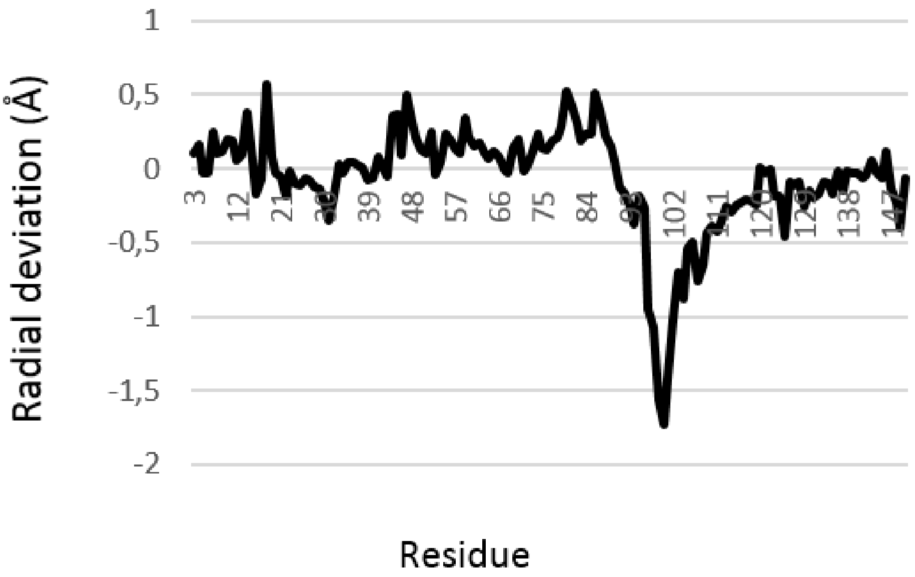

Supplementary Figure 2. Radial deviation between Ngb WT HP-310 and AP structures.

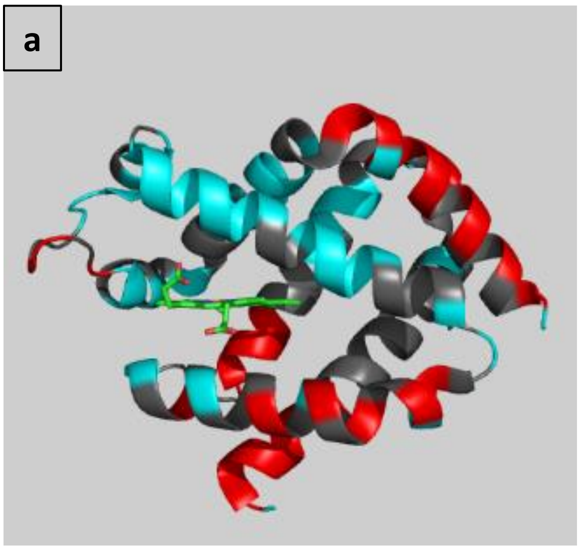

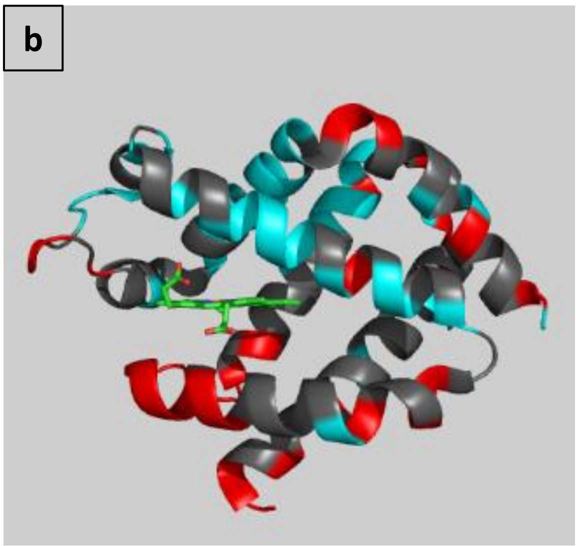

Supplementary Figure 3. B-factor differences between Ngb structures. Ngb shown in cartoon representation with the heme shown in stick representation. **A.** B-factor differences between WT HP-310 and WT HP-270 (on average 1.6 Å^2). Residues are colored according to the B-factor differences, in red if B-factor differences are higher than 4 Å^2, in cyan if B-factor differences have negative values, in grey otherwise. **B.** B-factor differences between V101F HP-240 and V101F AP (on average 10 Å^2). Residues are colored according to the B-factor differences, in red if B-factor differences are higher than 15 Å^2, in cyan if B-factor differences are lower than 5 Å^2, in grey otherwise.

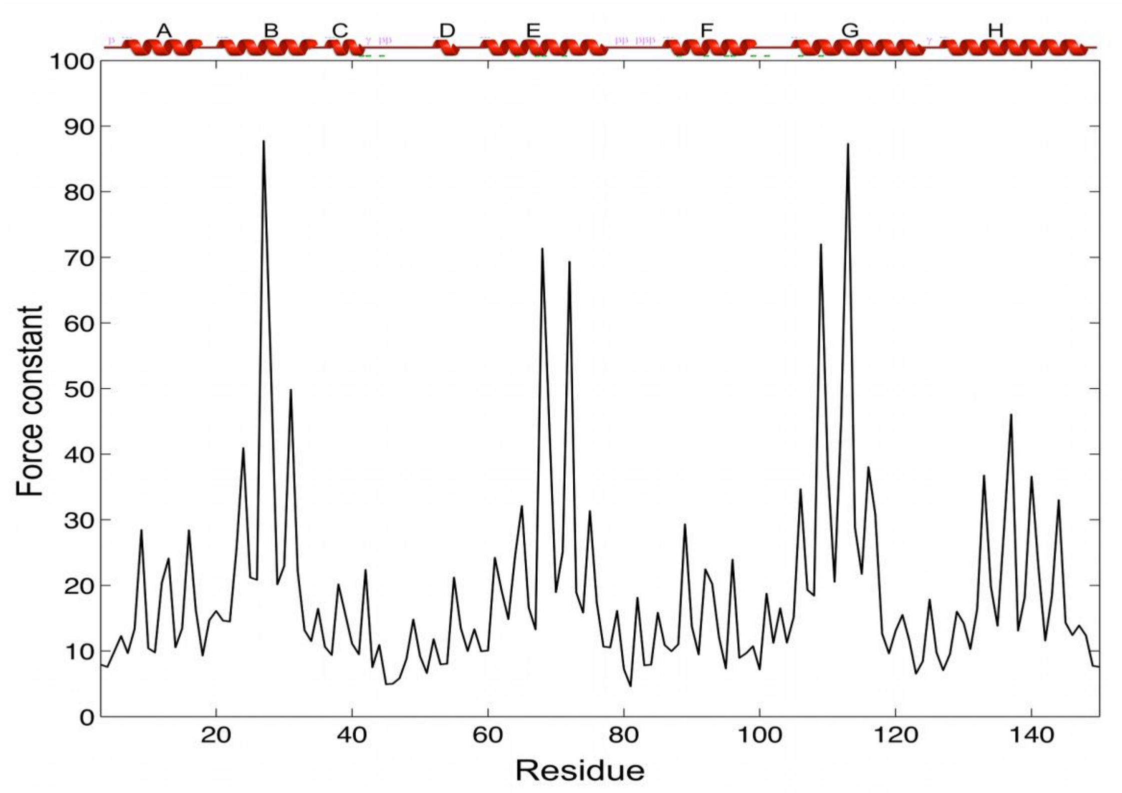

Supplementary Figure 4. Ngb WT mechanical profile at ambient pressure.
